# Comment on: Late Cachexia or treatment-related muscle wasting in long-term HNSCC survivors?

**DOI:** 10.1016/j.clinsp.2026.101002

**Published:** 2026-06-30

**Authors:** Erkan Topkan, Efsun Somay, Ugur Selek

**Affiliations:** a Department of Radiation Oncology, Faculty of Medicine, Baskent University, Adana, Turkey; b Department of Oral and Maxillofacial Surgery, Faculty of Dentistry, Baskent University, Ankara, Turkey; c Department of Radiation Oncology, School of Medicine, Koc University, Istanbul, Turkey

**Keywords:** Head and neck cancer, Cachexia, Chemoradiotherapy

Dear Editor,

We read with great interest the article by das Neves et al.,^1^ which aimed to evaluate the prevalence of cachexia among 120 Head and Neck Squamous Cell Carcinoma (HNSCC) patients treated with cisplatin based Chemoradiotherapy (CRT) with curative intent and presenting no evidence of disease. The authors hypothesized that HNSCC patients may continue to exhibit features consistent with cachexia even after long-term follow-up following curative-intent treatment. The authors should be commended for focusing on long-term, disease-free HNSCC survivors, a population that remains underrepresented in cachexia research despite its growing clinical relevance. The study provides valuable descriptive data suggesting that a subset of long-term HNSCC survivors exhibit persistent muscle depletion and functional impairment several years after completion of CRT. In particular, the assessment of anthropometric muscle measures and physical performance offers useful insight into muscle-related abnormalities observed in this survivor cohort. Nevertheless, several methodological and conceptual issues warrant clarification, as they have direct implications for the interpretation and generalizability of the reported findings.

A central concern relates to sampling and selection bias. Of 1940 screened patients, only 120 (≈6%) were ultimately included. Eligibility required survival beyond two years, absence of disease, regular follow-up, and willingness to participate. This survivor-enriched selection inherently favors patients with superior baseline prognosis, treatment tolerance, and functional reserve. Patients with early disease recurrence were explicitly excluded, and individuals experiencing severe toxicities or treatment-related complications are likely underrepresented due to survivorship and follow-up requirements. Consequently, the reported prevalence of cachexia cannot be extrapolated to the broader population of patients treated with curative CRT, nor to those who relapse or die earlier. Instead, the findings are most applicable to a highly selected subgroup of long-term survivors, which limits the ability to draw population-level inferences about cachexia risk and burden in the general HNSCC patient cohort.

In addition, the small absolute number of cachectic patients, particularly when stratified by diagnostic criteria (n = 10 using Evans criteria), introduces substantial sampling variability. Estimates derived from such small subgroups are statistically fragile and sensitive to minor misclassification, increasing the risk of false-negative and unstable association estimates. The limited sample size also restricts statistical power to detect true differences or associations and may result in wide confidence intervals, even when point estimates suggest potential effects. This limitation is especially relevant when interpreting null findings, such as the lack of association between cachexia and dysphagia or biochemical markers, which may reflect insufficient power rather than a true absence of association.

Another key issue concerns the use of two cachexia definitions without a formal comparative analysis. The authors apply both the Fearon and Evans criteria in parallel, reporting markedly different prevalence estimates (20.7%vs. 8.6%). However, no concordance analysis, overlap assessment, or reclassification evaluation is provided to clarify how frequently patients meet one, both, or neither set of criteria. Such analyses are essential to determine whether the two definitions identify overlapping or distinct patient subgroups and to elucidate the clinical relevance of discordant classification. Without this comparative framework, the clinical and biological meaning of meeting one definition but not the other remains unclear, thereby limiting interpretability. Given that the Fearon criteria emphasize weight loss and body composition, whereas the Evans criteria incorporate muscle strength and metabolic abnormalities, the observed divergence likely reflects the capture of biologically distinct phenotypes rather than measurement variability. A formal comparison, including cross-tabulation of criteria and evaluation of associated clinical features, would strengthen the study’s conclusions and enhance clinical applicability.

Importantly, most cachectic patients demonstrated normal hemoglobin, albumin, and C-reactive protein levels, indicating no clinically detectable systemic inflammatory or metabolic cachexia signature based on established diagnostic markers. This biochemical profile contrasts with classic cancer cachexia, which is typically characterized by inflammatory activation and metabolic disruption. In this context, labeling the observed phenotype as “cancer cachexia” may be conceptually problematic and potentially misleading. Contemporary frameworks increasingly distinguish active cancer cachexia ‒ driven primarily by tumor-induced inflammation and metabolic alterations ‒ from treatment-related muscle wasting, sarcopenia, or accelerated aging phenotypes observed in long-term cancer survivors.^2^, ^3^, ^4^, ^5^ The findings reported by das Neves et al. are therefore more consistent with a late toxicity of CRT, manifesting as chronic muscle loss and functional impairment, than with persistent cachexia biology mediated by ongoing tumor-host interaction. This distinction is crucial for both clinical interpretation and future research, as it shifts emphasis toward survivorship-oriented rehabilitation and long-term supportive care strategies.

Lastly, the cross-sectional design precludes meaningful causal inference regarding the origins or trajectory of muscle depletion in this cohort. It remains unclear whether the observed muscle loss primarily reflects residual treatment toxicity, late-onset metabolic alterations, or pre-existing vulnerability that may have contributed to long-term survival. The absence of pre-treatment or serial post-treatment muscle mass measurements further limits the ability to determine whether muscle depletion developed during therapy, emerged as a delayed effect, or persisted from baseline. Longitudinal imaging-based assessments, particularly CT-derived skeletal muscle indices obtained at multiple time points, would be required to disentangle these possibilities. Such data would enable characterization of temporal patterns of muscle change, facilitate more precise alignment with established sarcopenia and cachexia constructs,^6^, ^7^, ^8^ and inform the development of targeted prevention and rehabilitation strategies.

Despite these limitations, the study raises an important and underexplored clinical question: how should persistent muscle loss and functional impairment in disease-free HNSCC survivors be conceptualized, monitored, and managed. While the findings provide hypothesis-generating insights into late muscle abnormalities after curative CRT, they should be interpreted cautiously given the survivor-enriched study population, limited sample size, and absence of formal comparison between cachexia definitions. Addressing these challenges in future studies will require longitudinal, population-representative cohorts incorporating standardized muscle imaging and careful phenotypic distinction between cancer cachexia, sarcopenia, and treatment-related muscle wasting. Such efforts to clarify these issues would strengthen clinical interpretability and help advance a more precise, survivorship-oriented framework for muscle dysfunction in long-term HNSCC survivors. Finally, we thank the authors and Clinics for the opportunity to engage in constructive discussion of this otherwise valuable contribution to the field.

## Ethical approval

Not applicable.

## Data availability statement

Not applicable.

## Funding

No specific funding was received for this correspondence.

## Declaration of competing interest

The authors declare no conflicts of interest.
